# Pharmacokinetics of Oral Levonorgestrel in Women After Roux-en-Y Gastric Bypass Surgery and in BMI-Matched Controls

**DOI:** 10.1007/s11695-020-04447-x

**Published:** 2020-02-07

**Authors:** Charlotte Ginstman, Helena Kopp Kallner, Johanna Fagerberg-Silwer, Björn Carlsson, Andreas Ärlemalm, Ylva Böttiger, Jan Brynhildsen

**Affiliations:** 1 Department of Obstetrics and Gynecology, Linköping University, University Hospital, 58185 Linköping, Sweden; 2 Department of Clinical and Experimental Medicine, Linköping University, University Hospital, 58185 Linköping, Sweden; 3 Department of Obstetrics and Gynecology, Karolinska Institutet, 17177 Stockholm, Sweden; 4 Department of Women’s and Children’s Health, Karolinska Institutet, 17177 Stockholm, Sweden; 5 Department of Obstetrics and Gynecology, Danderyd Hospital, 18288 Stockholm, Sweden; 6 Department of Clinical Pharmacology, Linköping University, 581 85 Linköping, Sweden; 7 Department of Medical and Health Sciences, Linköping University, 581 85 Linköping, Sweden; 8 Division of Drug Research, Linköping University, 58185 Linköping, Sweden

**Keywords:** Gastric bypass surgery, Levonorgestrel, Obesity, Oral contraceptives, Pharmacokinetics

## Abstract

**Background:**

Women are advised to primarily use non-oral contraceptive alternatives after Roux-en-Y gastric bypass since it is not known if the surgery affects the pharmacokinetics of oral contraceptives.

**Methods:**

This is a multi-center, open label, phase 2 pharmacokinetic study performed at the University Hospital of Linköping and the Clinical Trials Center, Department of Obstetrics and Gynecology, Danderyd Hospital, Karolinska Institutet, Stockholm, Sweden. Fifteen women aged 18–40 years who had previously undergone Roux-en-Y gastric bypass surgery and reached a BMI < 30 were included. Fifteen BMI-matched women with no previous history of Roux-en-Y gastric bypass surgery served as a control group. After administration of a single dose of a combined oral contraceptive containing 0.03 mg ethinylestradiol/0.15 mg levonorgestrel, serum levonorgestrel concentrations were determined during a 24-h period using ultra performance liquid chromatography/tandem mass spectrometry. The area under the plasma concentration time curve of levonorgestrel (AUC_0–24h_) was the main outcome measure.

**Results:**

There were no significant differences in the studied pharmacokinetic parameters, AUC_0–24h_, total AUC, peak serum concentration (*C*_max_), time to peak serum concentrations (*T*_max_), apparent oral clearances of levonorgestrel (CL_oral_), or terminal half-lives (*t*½) between the groups.

**Conclusion:**

This is to our knowledge the first study to evaluate the pharmacokinetics of oral levonorgestrel in women with a BMI < 30 at least 1 year after RYGB compared with a BMI-matched group of women. We could not find any significant pharmacokinetic differences between the groups, suggesting that oral levonorgestrel may be used in non-obese women after Roux-en-Y gastric bypass once a stable body weight has been reached.

**Clinical Trial Number:**

EudraCT 2014–004677-17.

## Introduction

It is recommended that women avoid pregnancy for at least 12 months after bariatric surgery [1, 2]. This is to promote optimal postoperative weight loss and to avoid a potential risk of small-for-gestational-age infants and intrauterine growth restriction after bariatric surgery [3]. During recent years, there has been a change in bariatric surgery methods. Today, sleeve gastrectomy is the most common bariatric procedure worldwide [4, 5]. In 2016, however, RYGB was still performed in 30% of all worldwide bariatric procedures [4]. In 2017, RYGB still constituted 50.4% of all bariatric surgery in Sweden, although it had decreased rapidly from 97.5% in 2011 [6].

Changes in drug disposition may occur after RYGB [7]. There seem to be different effects of RYGB on different drugs. A decrease in the area under the plasma concentration time curve (AUC) of sertraline, duloxetine, and escitalopram has been shown after RYGB [8–10]. In contrast, no clinically relevant pharmacokinetic changes have been shown for morphine, caffeine, tolbutamide, omeprazole, venlafaxine, or metoprolol after RYGB [11–14]. Evidence from previous pharmacokinetic studies is conflicting regarding the potential for reduction in plasma levels of estrogens and progestogens after jejunoileal bypass [15, 16]. In a recently published study from our group, we could not show any clinically significant changes in the pharmacokinetics of desogestrel (etonogestrel) in obese women before compared with after RYGB [17]. However, the sample was small, and the results need to be confirmed. Due to the obesity-related increased risk of venous thromboembolism [18–20], the guidelines of the European society of contraception and reproductive health care (ESC) as well as the Swedish guidelines state that combined oral contraceptives should only be used in exceptional cases in obese women (Medical Eligibility Criteria (MEC) 2) [21, 22].

In summary, there is sparse, low quality, and conflicting data regarding the efficacy of oral contraception in women following bariatric surgery. Current guidelines recommend that non-oral hormonal contraception should be used after RYGB because there might be an increased risk of contraceptive failure when using oral contraceptives due to these uncertainties [2, 23, 24].

The pharmacokinetics of the compounds in combined oral contraceptives (COC) have to our knowledge not yet been studied in non-obese women after RYGB and compared with non-obese, non-operated women. Levonorgestrel (LNG)-containing COC is the first method of choice according to the European Medicines Agency as well as the Swedish Medical Product Agency [21, 22]. The aim of this study was to investigate oral LNG pharmacokinetics in women who had undergone RYGB and reached a BMI < 30 and who consequently no longer had a weight-related relative contraindication to COC. Furthermore, we aimed to compare these results with the corresponding figures in BMI-matched, non-operated women as our hypothesis was that the total exposure of the drug, reflected by the AUC would be lower in a group of women who had undergone RYGB surgery compared with non-operated women.

## Materials and Methods

We performed a multi-center, open label, phase 2 pharmacokinetic study to investigate the pharmacokinetics of levonorgestrel in 15 women with BMI < 30 who had previously undergone RYGB. Fifteen non-operated, BMI-matched women served as control group. Informed consent was obtained from all individual participants included in the study. Swedish-speaking women aged 18–40 years who, in order to measure single dose pharmacokinetics, had not used any hormonal contraceptives for at least 1 month before inclusion or Depo-Provera within the past 12 months, were considered as eligible study subjects. The women in the RYGB group had undergone surgery more than 1 year before inclusion, whereas the control group consisted of non-operated BMI–matched women. In both groups we excluded women who were breast-feeding or who had been pregnant within the past 3 months. Women with lactose intolerance and daily smokers were excluded. Previous bilateral oophorectomy or hysterectomy as well as an undiagnosed vaginal bleeding was also considered as an exclusion criterion for participation. Women regularly consuming St John’s wort and grapefruit juice or using medications or substances known to affect the cytochrome P450 system were ineligible, since these substances can interact with the pharmacokinetics of levonorgestrel. All participants had a normal gynecological examination including normal-appearing ovaries on a baseline sonogram with a 7.5-MHz transvaginal probe.

After ingesting one dose of 0.03 mg ethinylestradiol (EE) and 0.15 mg LNG (Neovletta, Bayer, Berlin, Germany), blood samples were taken from each study participant at 0, 0.5, 1, 1.5, 2, 2.5, 3, 4, 6, 8, 12, and 24. The protocol followed the same procedure as has been previously described (20). Briefly, the study medication was provided by the hospital research pharmacy (lot 54471c). The research nurses at the Clinical Trials Center, Department of Obstetrics and Gynecology, Danderyd Hospital, and at the Department of Clinical Pharmacology, University Hospital of Linköping, Sweden, performed all blood sampling. The study medication was ingested at 8:00 in the morning. All study participants were served standardized food according to a dietician’s recommendations. A calibrated weight scale was used for all body weight measurements. Thirty minutes after collection, the blood samples were centrifuged at 1970*g* for 10 min. Until the time of analysis, the plasma samples were stored at − 70 °C.

We used a validated ultraperformance liquid chromatography/tandem mass spectrometry method (UPLC/MS-MS) to measure the serum concentrations of LNG. The method was developed at the Department of Clinical Pharmacology, University Hospital of Linköping, from a method previously described by Seaves et al. [25]; this method was used in a previous study comparing desogestrel in women before and after RYGB [17]. The present study was performed in a very similar design, but no participants or result overlap between the studies. For the present study, we only used the online sample preparation method that is described as supplement material in the previous study [17].

Three quality control samples were prepared with the final concentrations of 0.2, 2, and 6 ng/ml. Intra-assay coefficients of variation were between 5 and 6%, and the accuracy of the method varied between 86 and 89%. The lower limit of quantification was 0.05 ng/ml. From each patient’s first blood sample, we measured plasma-SHBG (sex hormone–binding globulin) concentrations, which was analyzed in a standardized manner by the accredited laboratory at the Department of Clinical Chemistry at the University Hospital of Linköping (SWEDAC 1342).

Pharmacokinetic evaluations were made in the same manner as for the previous study [17]. Time to peak serum concentration (*T*_max_) and peak concentrations (*C*_max_) were taken directly from the original data. We fitted serum concentration values of LNG using a non-compartmental approach. The AUC from t0 to t24 was calculated using linear trapezoidal approximation. The total AUC was extrapolated from the slope of the last four measurements in the concentration curve. Elimination half-lives (*t*½) for LNG were calculated as ln2/kz, in which kz is a parameter describing the linear terminal slope of the log concentrations of levonorgestrel. The apparent oral clearance of LNG was calculated as dose/AUC.

### Statistics

COCs containing 0.10 mg levonorgestrel yields acceptable inhibition of ovulation [26]. The AUC after a single dose of these COCs is approximately 40–50% lower than for a COC containing 0.15 mg LNG [27, 28]. Such a difference in AUC seems acceptable. A difference exceeding 50% was therefore considered as clinically significant. Consequently, the sample size was set after estimating that a clinically relevant difference between the groups would be about 50% lower AUC in the RYGB group. To show this difference with 80% power and a *p* value of 0.05, the sample size was estimated at 15 in each group.

Descriptive statistics, including the mean, SD, minimum and maximum, and frequency, were calculated as appropriate for the distribution. The level of significance for all statistical tests was set at *p* < 0.05. Since all the data showed normal distribution, the groups were compared by using independent *t* test. SPSS software version 25 (IBM, USA) was used.

## Results

Between June 2016 and February 2018, a total of 15 Caucasian women were included in each study group. Five of the totals of 30 participants reported comorbidities. Two in the RYGB group had asthma, and one had hypothyroidism. One woman in the control group was receiving treatment for depression, and one had coeliac disease in complete remission. The median age was higher in the RYGB group (32 vs 26 years, *p* > 0.05). There were no significant differences in weight or BMI (70.8 vs 71.3 kg, and 25.6 vs 25.1) (Table 1).

**Table 1:** Demographic and clinical characteristics and pharmacokinetic parameters of levonorgestrel in 15 patients and 15 controls

	Previous RYGB (n15)	Controls (n15)	*p* value
Age (years)			
Median (range) Mean ± SD	32 (23–40) 32.3 ± 5.2	26(21–40) 28.2 ± 6.5	*p* = 0.069
BMI (kg/m^2^)			
Mean ± SD	25.6 ± 2.5	25.1 ± 3.1.	*p* = 0.619
Min-max	22.2–29.1	19.5–29.4	
Weight (kg)			
Mean ± SD	70.8 ± 8.2	71.3 ± 9.1	*p* = 0.871
Min-Max			
AUC_0–24h_ (ng*h/ml)			
Mean ± SD	19.9 ± 7.1	17.0 ± 8.1	*p = 0.302*
Min-max	10.7–30.6	8.0–37.9	
AUC_0 → ∞_ (ng*h/ml)			
Mean ± SD	40.3 ± 19.1	29.6 ± 14.3	*p = 0.091*
Min-max	20.4–81.0	11.3–60.5	
*C*_max_ (ng/ml)			
Mean ± SD	3.34 ± 1.16	2.96 ± 1.17	*p = 0.386*
Min-max	1.56–5.25	1.47–5.44	
C_24h_ (ng/ml)			
Mean ± SD	0.47 ± 0.2	0.37 ± 0.2	*p = 0.181*
Min-Max	0.23–0.85	0.14–0.77	
*T*_max_ (h)			
Median	1	1	
Range	(0.5–1.5)	(0.5–2)	
Mean ± SD	0.8 ± 0.3	1 ± 0.4	*p = 0.153*
*t*_½_ (hours)			
Mean ± SD	29.1 ± 14.6	24.0 ± 6.1	*p = 0.223*
CL_oral_ (L/h)			
Mean ± SD	8.5 ± 3.1	10.5 ± 4.3	*p = 0.157*
SHBG (ng/ml)			
Mean ± SD	87.5 ± 24.6	62.7 ± 22.7	*p = 0.008*
Min- max	47.7–141.4	27.3–111.4	

*AUC*_*0–24h*_ Area under the serum concentration time curves

*C*_*max*_ Peak serum concentrations,

*C*_*24h*_ Serum concentration 24 h after ingested dose of levonorgestrel

*T*_*max*_ Time to peak serum concentrations

*t*_*½*_ Terminal half-lives of levonorgestrel

*CL*_*oral*_ Apparent oral clearance of levonorgestrel

*SHBG* Sex hormone–binding globulin

Independent *t*-test was used for all statistical analyses

**Fig. 1:**
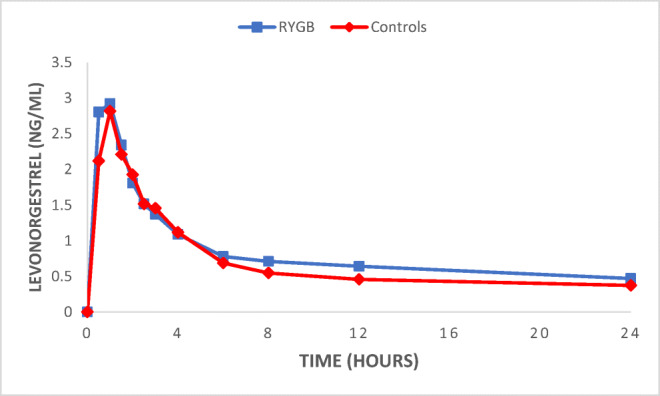
Mean values for the time course of levonorgestrel plasma concentration from 0 to 24 h (AUC_0–24_) for the 15 patients with a history of previous Roux-en-Y gastric bypass (RYGB) surgery and the 15 BMI-matched controls

**Fig. 2:**
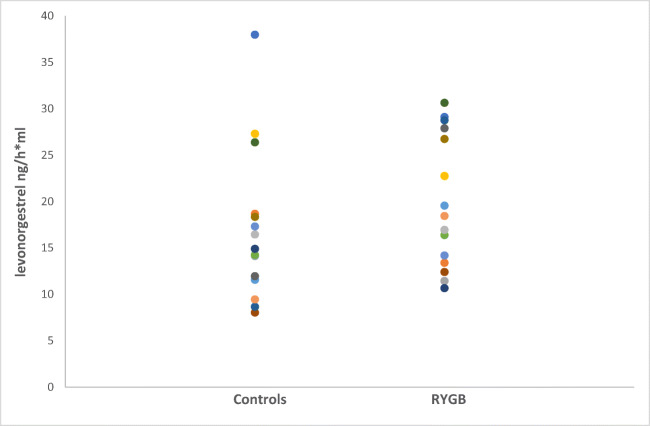
Individual values of area under the time concentration curves from 0 to 24 h (AUC_0–24_) after ingestion of a single dose 0.15 mg levonorgestrel in 15 patients with a history of previous Roux-en-Y gastric bypass (RYGB) and 15 BMI-matched controls

**Fig. 3:**
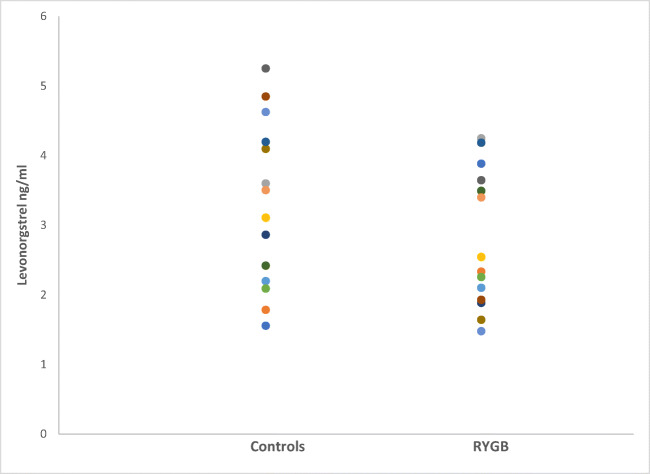
Individual values of peak serum concentrations (*C*_max_) after ingestion of a single dose 0.15 mg levonorgestrel in 15 patients with a history of previous Roux-en-Y gastric bypass (RYGB) and 15 BMI-matched controls

**Fig. 4:**
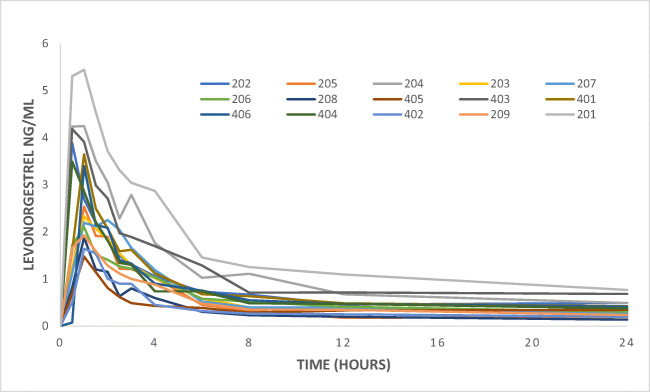
Individual values for the time course of levonorgestrel plasma concentration from 0 to 24 h (AUC_0–24_) for the 15 control patients

**Fig. 5:**
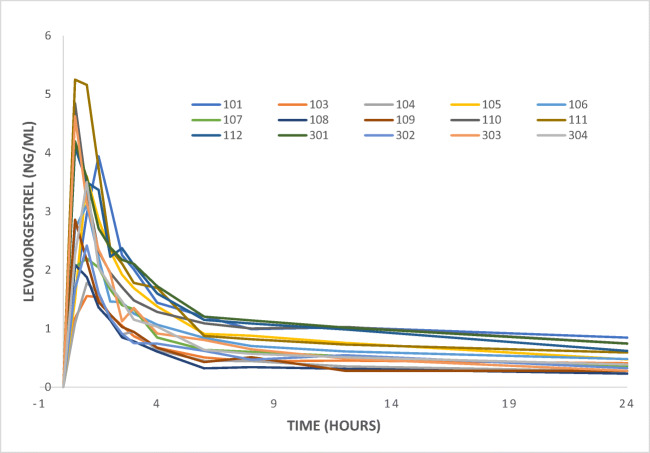
Individual values for the time course of levonorgestrel plasma concentration from 0 to 24 h (AUC_0–24_) for the 15 study patients after Roux-en-Y gastric bypass (RYGB) surgery

The mean AUC did not significantly differ between the groups (*p* = 0.302) (19.9 ± 7.1 ng h/ml in the operated group vs 17.0 ± 8.1 ng h/ml in the control group). A graphical view of the mean AUC is shown in Fig. 1. The total AUC (AUC_0 → ∞_) was not significantly different between the groups (*p =* 0.091). The intra-individual differences in AUC ranged from 10.7 to 30.6 ng h/ml in the RYGB group and from 8.0 to 37.9 ng h/ml in the control group (Table 1, Fig. 2). There was no significant difference between the groups in *C*_max_ (3.34 ± 1.16 ng/ml vs 2.96 ± 1.17 ng/ml). The individual *C*_max_ values are presented in Fig. 3 and a graphical presentation of the individual AUC in Figs. 4 and 5.

The result of the terminal half-life (*t*½), the trough value (C_24h_), the time to concentration max (*T*_max_), and the apparent oral clearance of LNG (CL_oral_) showed no significant difference between the groups. The concentration of SHBG was significantly higher in the RYGB group (*p* = 0.008). All results are shown in Table 1.

No serious adverse events were reported by any of the study participants. There were no adverse events related to the intake of the study drug.

## Discussion

To our knowledge, this is the first study comparing pharmacokinetic profiles in women who have undergone RYGB surgery with non-obese, non-operated women. Non-obese RYGB-operated women did not show any clinically relevant difference in the pharmacokinetic profile of LNG compared with BMI-matched non-operated women. Consequently, the results could not confirm our hypothesis, and RYGB does not seem to have any major effect on the pharmacokinetics of LNG in women with a BMI < 30. However, it must be emphasized that the results only are applicable for operated women who have reached a BMI < 30.

Although the number of patients in the present study was limited, other studies investigating the pharmacokinetic alterations of different substances after RYGB have similar sample sizes [13, 29]. We chose to include women at least 1 year after their RYGB in order to avoid analysis during the most rapid weight reduction. We studied a surgical method which is no longer the leading technique. However, this was not the situation at the time our study was planned. There are still a substantial number of fertile women who are still undergoing, or have undergone, RYGB, and needing contraceptive counseling and effective contraceptives. Previous studies have shown that women lack information on pregnancy planning and contraceptive use after bariatric surgery [30, 31].

For practical reasons, EE was not measured. Even though EE prevents the emergence of a dominant follicle through suppression of follicle-stimulating hormone secretion, it is the progestogen component of COCs that prevents ovulation [32], and consequently is the main contraceptive agent. Our study has focused only on the pharmacokinetics of LNG after RYGB. Since we found no significant differences, it seems reasonable to believe that the contraceptive efficacy after RYGB is unchanged.

Progestogens including LNG are assumed to be predominantly absorbed in the upper part of the gastrointestinal tract [33, 34]. The absorption could theoretically be affected by RYGB as it involves a bypass of the duodenum and part of the jejunum. Madden and coworkers showed in vitro metabolism of desogestrel in both the ileum and colon [35], indicating that the entire gut could be capable of absorption and first phase metabolism of progestins. The surface area in the gut is only one of several factors that may affect the pharmacokinetics of drugs after RYGB. Some drug transporters, metabolic enzymes, and efflux pumps occur with more frequency in the area being bypassed, which could affect the absorption of some substances [7]. In addition, gastric emptying, the intestinal transit time, and the pH in the ventricle may be of importance [7]. We were not able to control for all these factors. The concentrations of SHBG normally vary to a large extent between subjects, and the normal span is wide. There was a significant difference in the SHBG concentration of the two groups included in the present study. Theoretically this could imply that the women who had undergone RYGB would have a lower fraction of free, biologically active LNG. LNG is not bound only to SHBG but also to other plasma proteins as albumin. Unfortunately, we did not measure albumin. However, although SHBG was higher in the group of women who had undergone RYGB, we do not consider this difference as clinically relevant. All included women had values that were within the normal ranges, and in an everyday clinical practice, we do not take SHBG into account when considering efficacy of an oral contraceptive.

The majority of patients undergoing bariatric surgery are women of fertile age [6]. The current recommendations for women, however, are to avoid pregnancy for at least 12 months after surgery [2, 23]. Presently, however, there is little evidence supporting such recommendations; instead, they are based on caution. Previous studies have shown that many women of fertile age who have undergone bariatric surgery do not use contraceptives in the recommended way [31, 36]. This is probably at least partly explained by difficulties in finding an acceptable reliable method. Many women are recommended not to use oral methods because of a possible increased risk of contraceptive failure and may not be willing to use intrauterine contraception or implants. In a previous study of desogestrel and RYGB, no significant intra-individual differences could be seen pre- vs postoperatively [17]. Consequently, we have now studied the effects of RYBG on the pharmacokinetics of different progestogens both in- and between women and have found no differences. This strengthens the interpretation that oral contraceptives can be used in women who have undergone RYGB. A strength of this study is the homogenous group of women. This gives reliability of results as pharmacokinetic mechanisms such as metabolism and protein binding can differ among ethnic groups [37]. However, the ethnic homogeneity of the study may hamper generalizability.

Our study has focused only on the pharmacokinetics of LNG after RYGB. Since we found no significant differences it seems reasonable to believe that the contraceptive efficacy after RYGB is unchanged. The study is too small to evaluate pregnancy prevention effectiveness. Such study would be almost impossible to perform because the need of a very large population to yield an acceptable power.

Sivin et al. studied an LNG implant and found that steady state concentrations above 0.3 ng/ml were enough not to become pregnant [38]. We present trough values that reach this level even after one single dose. This implies that there is little or no impact of RYGB on the LNG efficacy as a contraceptive. The results of the pharmacokinetic parameters studied were in agreement with previously reported results [27, 39, 40]. There was a large intra-individual variation in peak serum concentration and AUC, but this was seen in both the RYGB and the control group and is in line with previously reported results [27, 41, 42].

In conclusion, this study could not reveal clinically significant differences in oral LNG pharmacokinetics in women with a BMI < 30 at least 1 year after RYGB compared with a BMI-matched group of women. This suggests that oral levonorgestrel may be used by non-obese women after a stable body weight has been reached.
